# *Vespa crabro* immunotherapy versus *Vespula*-venom immunotherapy in *Vespa crabro* allergy: a comparison study in field re-stings

**DOI:** 10.1186/s40413-018-0183-6

**Published:** 2018-02-02

**Authors:** Donatella Macchia, Gabriele Cortellini, Marina Mauro, Elisa Meucci, Oliviero Quercia, Mariangela Manfredi, Alessandro Massolo, Maurizio Valentini, Maurizio Severino, Giovanni Passalacqua

**Affiliations:** 1 Allergy and Clinical Immunology Unit, S. Giovanni di Dio Hospital, Florence, Italy; 2 Internal Medicine and Rheumatology Dept, Rimini Hospital, Rimini, Italy; 3 Allergy Unit, Sant’Anna Hospital, Como, Italy; 4 Allergy Unit, Internal Medicine Dept, Faenza, Italy; 5 Immunology and Allergy Laboratory, S.Giovanni di Dio Hospital, Florence, Italy; 6 Department of Biology, University of Pisa, Pisa, Italy; 7 Laboratoire Chrono-environnement UMR 6249 CNRS, Université Bourgogne Franche-Comté UsC INRA, Besancon, France; 8 Allergy and Respiratory Diseases, Policlinico San Martino, University of Genoa, L.Go R. Benzi 10, 16132 Genoa, Italy

**Keywords:** Hymenoptera venom allergy, Venom immunotherapy, *Vespa crabro*, Vespula, Safety, Efficacy

## Abstract

**Background:**

In ascertained allergic sensitization to *Vespa crabro* (VC) venom, the European guidelines still consider venom immunotherapy (VIT) with *Vespula* (VE) venom sufficient to achieve an adequate protection against VC. However, antigen 5 immunoblotting studies showed that a genuine sensitization to VC venom may exist. In such cases, a specific VC venom would be preferable for VIT treatment. Since in the last few years, VC venom extracts became available for diagnosis and desensitization, we assessed the efficacy and safety of VIT with a VC-VIT, compared to VE extract.

**Methods:**

Patients stung by VC, and carefully diagnosed for specific sensitization and indication to VIT underwent a 5-year course of immunotherapy with either VE or VC extracts*.* The severity of reactions at the first sting (pre-VIT) and after field re-stings (during VIT) were compared.

**Results:**

Eighty-three patients, treated with VE extract and 130 patients treated with VC extract completed the 5-year course of VIT. Only a fraction of those patients (43,8%) were field-re-stung by VC: 64 patients on VC VIT and 69 on VE VIT. In the VC VIT group, reactions at re-sting were: 50 negative, 12 large local reactions, 4 systemic reactions (Muller grade I). In this group the VC VIT efficacy was 93,8%. In the VE VIT treated group the reactions at VC re-sting were: 51 negative, 10 large local reactions and 9 systemic reactions (5 Muller I, 3 Mueller III, 1 Muller IV). In this group the overall efficacy of VIT was 87,0%. The difference in efficacy between the two groups was not statistically significant, as previously reported in literature. Nonetheless, field sting systemic reactions Muller III and IV were recorded only in those patients receiving VE VIT.

**Conclusion:**

This observation suggests that in patients with ascertained VC-induced allergic reactions a specific VC VIT, where available, would be more adequate, at least concerning the safety profile.

## Background

Despite relatively rare, Hymenoptera venom allergy (HVA) remains an important cause of severe reactions and even fatalities. The occurrence of insect sting-related mortality ranges between 0.03 and 0.48 fatalities per 1,000,000 individuals per year [1]. The main responsible of those events are the insects belonging to the families of Vespidae (including Vespinae and Polistinae), being *Vespula* and *Dolichovespula* the most frequent allergenic sources*.* Within the genus *Vespula* (VE) there are *Vespula germanica* and *Vespula vulgaris,* which are usually smaller in dimensions, and differ from *Dolichovespula* in some other morphological aspects for instance, *Dolichovespula* has a sagittaly more elonged head. In Europe, the most represented species are *Dolichovespula media, D. saxonica* and *D. sylvestris*. Within the genus *Vespa*, *Vespa crabro (VC)* (European hornet) is predominant [2]. Of note, also Polistes spp. are currently recognized as a relevant allergenic source [3].

The molecular composition of the venoms of the most represented species is known quite in detail. The most important vespid allergens are phospholipase A1, hyaluronidase, and antigen 5 (Ag-5). These antigens are present both in VE and in VC venoms [4]*.* Among vespids, venoms are usually widely cross-reactive. In fact, most of the VE allergens share a 95% homology [5, 6] in their aminoacid sequency. As a consequence, also diagnostic and therapeutic extracts may display a certain cross-reactivity [7, 8]. Also, there is a not negligible cross-reactivity among *Vespula*, *Vespa* and *Dolichovespula* venoms [9, 10].

Studies conducted in the Mediterranean area showed that the risk of developing severe systemic reactions is increased by about three-fold with VC, as compared to honeybee or VE species [11]. It can be hypothesized that some of the patients with HVA due to VC were previously sensitized by VE stings. Thus, in those subjects with an ascertained VC systemic reactions, the venom immunotherapy (VIT) with VE extracts would be sufficient to confer protection, and this is what the European guidelines suggest [12–14]. On the other hand, CAP-inhibition and immunoblotting-based studies showed that those techniques remained inconclusive in about 50% patients, suggesting that sensitization against VC Ag-5 is relevant and genuine [11]. The phylogenetic tree, concerning Ag-5, confirms that VE and VC are quite distant [15, 16].

Since a commercial extract for diagnosis and VIT for VC is now available in Italy (and can be used everywhere as named-patient product), we compared the efficacy and safety of VC and VE VIT in patients with ascertained reactions to VC. This was done directly evaluating the field re-stings during VIT.

## Methods

All the involved patients were recruited at various Allergy Centres in Italy (Como, Faenza, Firenze and Rimini) and received VIT according to the EAACI guidelines [11] and standard of care. Most patients, obviously, received VE VIT, since VC preparations were not available before 1990. Consecutive patients referred to our units for a previous systemic reaction following a VC sting (from to 1990 to 2016) were observed. Only patients who could recognize or document with certainty VC as the stinging hymenoptera were included. Systemic reactions were graded according to Mueller [17]. The diagnostic procedures were those recommended by the European Academy of Allergy and Clinical Immunology [11, 18]. These procedures included skin prick test, intradermal test and CAP-assay for honeybee, *Vespula* spp. (Alk, Hornsholm, Denmark; Stallergenes, Antony Cedex, France), *Polistes dominula* and *Vespa crabro* (Anallergo, Florence, Italy). In parallel, specific IgE by ImmunoCAP-assay (Phadia AB, Upssala, Sweden) for the mentioned allergenic sources and serum tryptase by ImmunoCAP (Phadia AB, Upssala, Sweden) were carried out. According to the results of diagnostic tests, clinical indications, judgement of physicians, and availability of the VC extract the patients were allocated to either VE VIT (Alk, Hornsholm, Denmark; Stallergenes, Antony Cedex, France) or VC (*Vespa Crabro*, Anallergo, Florence, Italy) VIT. A modified rush protocol was used in both the VC and VE groups. For VE VIT, an aqueous extract (100 mcg/mL) was used during build-up and maintenance. For VC VIT, an aqueous extract (100 mcg/mL) was used in the first two sessions, then a tyrosine-absorbed extract was given at maintenance (Table 1). During the first 3 sessions the escalating doses were given at 30-min intervals, until the 4th session, when the maintenance dose of 100 mcg/mL was administered. At maintenance, a 100 mcg/mL dose was given monthly for the first year, then every 6 or 8 weeks until the 5th year. On field re-sting reactions (number and severity) by VC were assessed and described in the two groups. If there was more than one re-sting without reactions in the same patient, only the first re-sting was counted and recorded.

**Table 1:** Build-up phase for rush VIT

Day	Concentration mcg/ml	Dose in mcg
1	0.1	0.01
	1	0.1
	1	1
	10	3
2	10	5
	100	10
	100	20
3	100	30
	100	35
	100	35
10	100	100

The dataset was aggregated by patient, and summary statistics for key variables were calculated for each single patient at the beginning and end of the immunotherapy (0 and 60 months). To compare the efficacy between the two therapies in reducing the reaction to subsequent stings, we considered only patients who completed the course of VIT (60 months) and were field re-stung by VC within this time frame. We also compared the most severe reactions at re-sting versus those reported at the initial diagnosis, and the most severe reactions to sting reported during the 5 years of the VIT course. Moreover, we compared the results from the IgE specific immunoassay and the skin test (in logarithmic scale) at time 0 and at the end of the therapy (after 60 months) using a General Linear Model (GLM) analysis for repeated measures. Sex ratio, age, specific IgE values, skin test results and worst reaction reported at baseline were compared between the two groups (VE vs VC) to assess the homogeneity of datasets at the beginning of VIT. Sex ratio, intradermal results to VC and systemic reactions by VC at baseline were compared using a Pearson chi-square analysis, whereas the other values were compared using the Student’s *t* test or Mann-Whitney tests for independent samples in the case of not homogeneous variance at the Welch’ test for variance homogeneity [19]. For all post-treatment univariate tests, we report both the one- and two-tails test, under the assumption that VC VIT could be more effective. The probability distributions for the Mann-Withney tests, as well as for the Chi-Square tests were estimated using a permutation (Exact) approach [20]. All statistical analyses were carried out using IBM® SPSS® Statistics Ver. 24.

## Results

Systemic reactions were graded according to Mueller [18]. In total, 303 patients (229 male, age range 10–78) (Table 2) with a systemic reaction due to ascertained VC sting were included. Of them, 83 received VE VIT and 220 VC VIT. There was no significant difference between the two groups at baseline, but for age and threshold intradermal reactivity to VC (Table 2).

**Table 2:** Characteristics of the population of patients receiving VE or VC VIT between 1990 and 2016. Mean, minimum and maximum values are reported along with the levels of significance for the Student’s (t), Mann-Whitney (MW) and chi-square (*X*^*2*^) tests. The significant differences are in bold

	Group *VC N* = 220	Group *VE N* = 83	Statistical differences
Age mean (range)	49.9 (10–78)	43.7 (15–74)	MW, ***P*** **< 0.014**
M/F	166/54	66/17	*X*^*2*^, *P* = 0.544
Grade before VIT n (%)			*X*^*2*^, *P*_*Exact*_ = 0.134
Mueller 1	13 (6%)	0 (0%)	
Mueller 2	30 (14%)	11 (14%)	
Mueller 3	64 (29%)	29 (34%)	
Mueller 4	111 (51%)	43 (52%)	
VC Intradermal test (thereshold in mcg/ml)^a^			*X*^*2*^, ***P*** **= 0.003**
1 mcg/ml	45 (20%)	26 (31%)	
0,1 mcg/ml	70 (32%)	34 (41%)	
0,01 mcg/ml	60 (27%)	20 (24%)	
0,001 mcg/ml	21 (10%)	3 (4%)	
0,0001 mcg/ml	24 (11%)		
VC specific IgE mean (range) kU/L	2.1 (0.3–73.8)	3.0 (0.2–32.9)	*t*, *P* > 0.05
Tryptase, mean (range)	5.5 (1–40)	5.2 (1.3–39.1)	*t*, *P* > 0.05

^a^lowest venom concentration able to induce at least a 5-mm diameter’s wheal with erythema

Among the considered population of 303 patients, 133 (43.8%) were re-stung by VC during the 5-year VIT course: 64 on VC VIT and 69 on VE VIT. The VE group patients totalized 99 on-field re-stings. We compared the worst reaction at baseline (before VIT), and the worst reaction at re-sting during VIT. In this group, during VIT there were: 51 no reaction, 10 large local reactions and 9 systemic reactions (5 grade Mueller I, 3 grade III, 1 grade IV) (Table 3). The VC group patients had a total of 98 re-stings: 48 no reaction, 12 large local reactions and 4 systemic reactions (all grade Mueller I) (Table 3). Of note, 93.8% (60/64) of patients treated with VC venom and 87.0% (60/69) of those treated with VE venom, were protected at re-sting. This difference was not statistically significant (*X*^*2*^ = 1.738, df = 1, *P*_*Exact(2-tails)*_ = 0.247, _*Exact(1-tail)*_ *=* 0.153).

**Table 3:** Outcome of field re-sting in 133 patients during the 5-year VE or VC VIT. Response severity before and during VIT are reported as absolute numbers

	VE VIT (*n* = 69)		VC VIT (*n* = 64)	
	Before	During	Before	During
NEG	0	50	0	48
LLR	0	10	0	12
Muller 1	0	5	3	4
Muller 2	9	0	7	0
Muller 3	25	3	21	0
Muller 4	35	1	33	0

When these relations where examined by the GLM approach, we detected an overall significant effect of immunotherapy (within subjects effect for VIT; *F* = 952.446, df = 1, *P* ≤ 0.001) with a significant reduction in the severity of the response to VC sting during VIT. On the other hand no significant effect between the different venoms (between subjects; *F*_*1, 118*_ = 0.043, *P* = 0.837), to the baseline values of intradermal reactivity to VC (between subjects; *F*_*1, 118*_ = 0.985, *P* = 0.323) or to specific IgE to VC (between subjects; *F*_*1, 118*_ = 0.979, *P* = 0.325), even when controlling for possible within subjects second-term interactions (all VIT interaction terms *P* ≥ 0.05).

No patient treated with VC venom had systemic reactions more severe than Mueller I, whereas in the VE group 5.8% (4/69) had Mueller III or IV reactions (Table 3). In this case, we detected an almost significant difference comparing the number of severe reactions out of the total number of reactions in the two groups (VE = 4/9, VC, 0/4; *X*^*2*^ = 2.568, df = 1, *P*_*Exact(2-tails)*_ = 0.120, *P*_*1-tail*_ = 0.069), particularly assuming a 1-tail test, given the expectation is that the VC vaccine is more effective than the VE one. Similarly, when comparing mild reactions (large local + Mueller 1) and severe reactions (Mueller 2–4) in the two groups an almost significant trend was seen (VE, 15/4; VC, 16/0; *X*^*2*^ = 3.803, df = 1, *P*_*Exact(2-tails)*_ = 0.109, *P*_*Exact(1-tail)*_ = 0.074) clearly due to the small sample size.

There was no severe adverse event due to VIT treatments: only LLRs occurred during the build-up phase, not interfering with the administration schedule. Finally, the safety of the VC VIT was confirmed as safe as for other preparations [10, 14].

## Discussion

VC stings are frequently at risk for severe or life-threatening reactions in sensitized subjects [10]. Usually, in patients with ascertained systemic reactions from VC*,* VIT with VE extracts is considered effective and safe. Nonetheless, some patients may have a sensitization to epitopes of VC allergens [21] that are not covered by the standard VE extracts, as shown by mass spectrometry analyses [22]. As a matter of fact, in a previous study involving 202 patients with VC sting and treated with VE VIT [13], the use of VE VIT was justified by the fact that the majority of patients would be not have previously been sensitized by VE stings. In this study, after field resting, out of 8 stings 5 were negative, one had a large local, and two systemic reactions.

The present study, by assessing in prospective way the characteristics of field re-stings, showed that both VE and VC VIT are equally effective, thus overall confirming the data reported in literature so far. Nonetheless, reactions Mueller III and Muller IV grade at field re-sting were observed only in patients treated with VE-VIT; this suggests the hypothesis that in patients with VC sting systemic reactions, a VC-VIT might be more adequate. The difference was not formally significant, but this can be ascribed to the small number of patients who actually had severe reactions (*n* = 13). It is true that the study was not randomized, but populations were homogeneous, and the same diagnostic procedures were used in all patients. In addition this model is quite “clean” since VC can be easily distinguished from other species, due to the dimensions and the essentially nocturnal habits. A gap still exists in the diagnostic approach since, at present, recombinant molecules for diagnosis are available for Ag-5 of VE and polistes, but not for VC, which partly differ from the other species [14, 23]. So far, the literature about VC allergy remains poor [23]. Probably, the introduction of diagnostic preparation more specific for the various species will further refine the prescription of VIT.

## Conclusion

In conclusion, our results suggest that in patients with ascertained VC systemic reactions, a VC-specific VIT, where available, might be more adequate at least from the safety viewpoint. Where VC VIT is not commercially available, the VE VIT can be used, with a comparable efficacy. In addition, the safety of the VC VIT preparation herein described is comparable to that of the VE preparations, during the rush build-up phase. Of course, future studies including larger samples are required.

## Abbreviations

HVA: Hymenoptera venom allergy
VC: *Vespa crabro*
VE: *vespula*
VIT: Venom immunotherapy

## References

[1] Antonicelli L, Bilo MB, Bonifazi F (2002). Epidemiology of hymenoptera allergy. Curr Opin Allergy Clin Immunol.

[2] Turillazzi S (2013). Allergologia e Dermatologia Entomologiche.

[3] Sturm GJ, Varga EM, Roberts G, Mosbech H, Bilò MB, Akdis CA. EAACI guidelines on allergen immunotherapy: hymenoptera venom allergy. Allergy. 2017; 10.1111/all.13262. 10.1111/all.13262 28748641

[4] Spillner E, Blank S, Jakob T (2014). Hymenoptera allergens: from venom to “venome”. Front Immunol.

[5] Sastre J (2010). Molecular diagnosis in allergy. Clin Exp Allergy.

[6] Chugo S, Lizaso MT, Alvarez MJ, Arroabaren E, Lizarza S, Tabar AI (2015). Vespa Velutina nigritorax: a new causative agent in anaphylaxis. J Investig Allergol Clin Immunol.

[7] King TP, Alagon AC, Kuan J, Sobotka AF, Lichtenstein LM (1983). Immunochemical studies of yellow jacket venom proteins. Mol Immunol.

[8] Wicher K, Reisman RE, Wypych J, Elliott W, Steger R, Mathews RS, Arbesman CE (1980). Comparison of the immunogenicity of venoms of various species of yellow jackets (genus Vespula). J Allergy Clin Immunol.

[9] Hoffman DR (1986). Allergens in hymenoptera venoms XVI. Studies of the structures and cross-reactivities of vespid venom phospholipases. J Allergy Clin Immunol.

[10] Panzani R, Blanca M, Sanchez F, Juarez C (1994). Sensitivity to European wasps in a group of allergic patients in Marseille: preliminary results. J Investig Allergol Clin Immunol.

[11] Antonicelli L, Bilò MB, Napoli G, Farabollini B, Bonifazi F (2003). European hornet (Vespa Crabro) sting: a new risk factor for life-threatening reaction in hymenoptera allergic patients?. Allerg Immunol (Paris).

[12] Bonifazi F, Jutel M, Bilò MB, Birnbaum J, Muller U, the EAACI Interest Group on Insect Venom Hypersensitivity (2005). Prevention and treatment of hymenoptera venom allergy: guidelines for clinical practice. Allergy.

[13] Kosnik M, Korosec P, Silar M, Music E, Erzen R (2002). Wasp venom is appropriate for immunotherapy of patients with allergic reaction to the European hornet sting. Croat Med J.

[14] Alfaya Arias T, Soriano Gómis V, Soto Mera T, Vega Castro A, Vega Gutiérrez JM, Alonso Llamazares A, Antolín Amérigo D, Carballada Gonzalez FJ (2017). Hymenoptera allergy committee of the SEAIC. Key issues in hymenoptera venom allergy: an update. J Investig Allergol Clin Immunol.

[15] Severino MG, Caruso B, Bonadonna P, Labardi D, Macchia D, Campi P, Passalacqua G (2010). Cross reactivity between European hornet and yellow jacket venoms. Eur Ann Allergy Clin Immunol.

[16] Pantera B, Hoffman DR, Carresi L, Cappugi G, Turillazzi S, Manao G, Severino M, Spadolini I, Orsomando G, Moneti G, Pazzagli L (2003). Characterization of the major allergens purified from the venom of the paper wasp Polistes Gallicus. Biochim Biophys Acta.

[17] Mueller HL (1966). Diagnosis and treatment of insect sensitivity. J Asthma Res.

[18] Bilò MB, Rueff F, Mosbech H, Bonifazi F (2005). Oude-Elberink JNG and the EAACI interest group on insect venom hypersensitivity. Allergy.

[19] Sokal RR, Rohlf FJ (1995). Biometry. The principles and practice of statistics in biological research.

[20] Good P (2000). Permutation tests: a practical guide to resampling methods for testing hypotheses.

[21] Blanca M, Garcia F, Miranda A, Carmona MJ, Garcia J, Fernandez J, Terrados S (1991). Determination of IgE antibodies to Polistes Dominulus, Vespula Germanica and Vespa Crabro in sera of patients allergic to vespids. Allergy.

[22] Turillazzi S, Bruschini C, Lambardi D, Francese S, Spadolini I, Mastrobuoni G (2007). Comparison of the medium molecular weight venom fractions from five species of common social wasps by MALDI-TOF spectra profiling. J Mass Spectrom.

[23] Golden DBJ (2005). Insect sting allergy and venom immunotherapy: a model and a mystery. Allergy Clin Immunol.

